# Editorial: Metal homeostasis in microbial physiology and virulence

**DOI:** 10.3389/fcimb.2023.1183137

**Published:** 2023-03-22

**Authors:** José F. da Silva Neto, Charley C. Staats, Mauricio H. Pontes

**Affiliations:** ^1^ Departamento de Biologia Celular e Molecular e Bioagentes Patogênicos, Faculdade de Medicina de Ribeirão Preto, Universidade de São Paulo, Ribeirão Preto, Brazil; ^2^ Programa de Pós-Graduação em Biologia Celular e Molecular, Centro de Biotecnologia, Universidade Federal do Rio Grande do Sul, Porto Alegre, Brazil; ^3^ Department of Pathology and Laboratory Medicine, Penn State College of Medicine, Hershey, PA, United States; ^4^ Department of Microbiology and Immunology, Penn State College of Medicine, Hershey, PA, United States

**Keywords:** transition metals, metal acquisition systems, metal efflux systems, nutritional immunity, siderophores, metalloregulators, bacterial and fungal virulence, iron homeostasis

Biological-relevant transition metals such as iron, zinc, manganese, and copper are at the center of a battle between pathogens and hosts, playing a major role in the outcome of bacterial and fungal infections. Hosts withhold metals from invading microbes or attempt to intoxicate them with metal overload. Microbes may employ numerous strategies to circumvent these host-imposed nutritional immunity mechanisms and ensure an appropriate metal supply for their physiological demands. In this Research Topic, a series of Review and Original Research Articles presents distinct facets of metal homeostasis in the context of microbe-host interactions.

*Mycobacterium tuberculosis*, one of the most important human pathogens, causes millions of tuberculosis cases worldwide. Rodriguez et al. provided an overview of the response of *M. tuberculosis* to changes in iron availability, emphasizing the relevance of iron to tuberculosis pathogenesis. The authors summarize the multiple mechanisms used by *M. tuberculosis* for iron uptake, focusing on the pathways involving the siderophores mycobactin and carboxymycobactin. The impact of iron limitation on many other aspects of *M. tuberculosis* physiology is also discussed, including upregulation of virulence factors, modification of the cell surface, increase of extracellular vesicle release, and metabolic switches toward quiescent, antibiotic tolerant states. Given the prominent role of iron for *M. tuberculosis* virulence, the authors suggest that key proteins involved in iron utilization could be new targets for therapeutic intervention. The Cornelissen group contributes two interesting Reviews on metal homeostasis in *Neisseria meningitidis* and *Neisseria gonorrhoeae*, two human-specific pathogens adapted to steal metals directly from host metal-binding proteins. Branch et al. provide a broad perspective of how these two pathogenic *Neisseria* that cause so distinct human diseases have evolved similar mechanisms to manage zinc, manganese, and copper in the context of human infections. The authors emphasize how the human host restricts these metals in the infection sites by increasing the release of calprotectin and how the pathogenic *Neisseria* sense and respond to such alterations by expressing surface transporters that pirate zinc from calprotectin. The authors also discuss the emergent field of host-mediated metal intoxication in the context of *Neisseria* infections. Stoudenmire et al. present a focused review of how *N. gonorrhoeae*, the bacterium that causes gonorrhea, bypasses human nutritional immunity by utilizing iron from host metalloproteins. As a pathogen unable to synthesize its own siderophores, *N. gonorrhoeae* relies on multiple iron-regulated TonB-dependent transporters to steal iron directly from host proteins such as hemoglobin (HpuAB), transferrin (TbpA), and lactoferrin (LbpA).

Heme is an abundant iron reservoir in humans, and many pathogens can use heme from host hemoproteins like hemoglobin. de Lima et al. established that the ChuPRSTUV system is required for heme and hemoglobin utilization in *Chromobacterium violaceum*, a Gram-negative environmental bacterium that causes severe human infections. Using a mouse model of acute infection, the authors demonstrate that *C. violaceum* requires the combined activities of Chu and its siderophores to acquired iron and display full virulence. Interestingly, the authors also discovered that the ChuP protein regulates siderophore utilization, providing an integrated mechanism by which *C. violaceum* may control iron acquisition *via* siderophore and heme during infection. Unlike *C. violaceum*, the obligate human pathogen *Streptococcus pyogenes* prefers heme as an iron source. Lyles et al. investigated the role of the protein HupZ in the heme metabolism of *S. pyogenes*. They showed that HupZ binds heme, but it has a weak heme degradation activity, suggesting that this protein may function as a heme chaperone and/or detoxifying protein. Using a murine model of vaginal infection, the authors demonstrated that HupZ contributes to *S. pyogenes* colonization of the host.

Two Original Research Articles reported particular aspects of the host nutritional immunity in response to bacterial infection. Grubwieser et al. investigated how alveolar epithelial cells adapt their cellular iron homeostasis in response to *in vitro* infection by *Klebsiella pneumoniae* and *Escherichia coli*, two species that frequently cause hospital-acquired pneumonia. The authors found that the extracellular pathogen *E. coli* induces an iron retention phenotype in A549 cells. In contrast, infection by the facultative intracellular bacterium *K. pneumoniae* promotes an iron export phenotype. These findings suggest that human alveolar cells can employ distinct iron-based nutritional immunity mechanisms as a defense against invading pathogenic bacteria. Baishya et al. explored novel roles of calprotectin in the biofilms of *Pseudomonas aeruginosa* and *Staphylococcus aureus*, during growth in laboratory culture medium and in a mouse model of chronic wound infection. Whereas the innate immune protein calprotectin has been primarily studied in the context of its metal chelating activities, little is known about its metal-independent antimicrobial activity. Using a number of microscopy techniques, the authors found that calprotectin stimulates bacterial encapsulation in mesh-like structures, an effect that seems to be independent of calprotectin’s ability to bind metals. These findings provide new clues about how calprotectin inhibits bacterial growth.

Metal homeostasis in the context of fungal-host interaction is the theme of two Original Research Articles. Souza et al. investigated the exoproteome of the fungus *Paracoccidioides brasiliensis* in response to iron deprivation by mass spectrometry. In previous works, the group has demonstrated that this human pathogen responds to iron deprivation by increasing production of siderophores and the activity of cell surface associated ferric reductases. In the current work, the authors identified 141 proteins, 64 of which were predicted to be secreted. The exoproteome data was validated by the demonstration that Cyb5 is a secreted iron-binding protein. Another important fungal pathogen, the airborne human mold *Aspergillus fumigatus*, has complex systems for maintaining copper homeostasis, which are required for *A. fumigatus* pathogenicity. Yap et al. reported that the availability of certain amino acids (especially histidine) and proteins increases resistance to copper in *A. fumigatus*. The authors provide several lines of evidence that the mechanism of protection involves histidine inhibition of low-affinity copper acquisition systems by extracellular copper complexation, and demonstrate that iron limitation decreases copper resistance. As copper resistance of *A. fumigatus* is crucial to its survival during infection, this work provides novel insights into how this pathogen may avoid nutritional immunity strategies that are imposed by the host.

This Research Topic sheds light on the complex mechanisms used by bacterial and fungal pathogens to scavenge essential metals from their hosts, as well as how the host can employ nutritional immunity to restrict access to these metals. By understanding such mechanisms, we will be able to develop new strategies to combat these pathogens and reduce the prevalence of diseases they cause.

## Author contributions

JN, CS, and MP edited the topic and wrote the manuscript. All authors contributed to the article and approved the submitted version. 

